# Ginsenoside Rh2 shifts tumor metabolism from aerobic glycolysis to oxidative phosphorylation through regulating the HIF1-α/PDK4 axis in non-small cell lung cancer

**DOI:** 10.1186/s10020-024-00813-y

**Published:** 2024-04-26

**Authors:** Xiyu Liu, Jingjing Li, Qingqing Huang, Mingming Jin, Gang Huang

**Affiliations:** 1 Shanghai University of Traditional Chinese Medicine, 201203 Shanghai, P.R. China; 2 Shanghai Key Laboratory of Molecular Imaging, Shanghai University of Medicine and Health Sciences, 279 Zhouzhu Road, Pudong New Area, 201318 Shanghai, China

**Keywords:** Ginsenoside Rh2, NSCLC, PDK4, Glycolysis, Oxidative phosphorylation

## Abstract

**Background:**

Ginsenoside Rh2 (G-Rh2), a steroidal compound extracted from roots of ginseng, has been extensively studied in tumor therapy. However, its specific regulatory mechanism in non-small cell lung cancer (NSCLC) is not well understood. Pyruvate dehydrogenase kinase 4 (PDK4), a central regulator of cellular energy metabolism, is highly expressed in various malignant tumors. We investigated the impact of G-Rh2 on the malignant progression of NSCLC and how it regulated PDK4 to influence tumor aerobic glycolysis and mitochondrial function.

**Method:**

We examined the inhibitory effect of G-Rh2 on NSCLC through I proliferation assay, migration assay and flow cytometry in vitro. Subsequently, we verified the ability of G-Rh2 to inhibit tumor growth and metastasis by constructing subcutaneous tumor and metastasis models in nude mice. Proteomics analysis was conducted to analyze the action pathways of G-Rh2. Additionally, we assessed glycolysis and mitochondrial function using seahorse, PET-CT, Western blot, and RT-qPCR.

**Result:**

Treatment with G-Rh2 significantly inhibited tumor proliferation and migration ability both in vitro and in vivo. Furthermore, G-Rh2 inhibited the tumor’s aerobic glycolytic capacity, including glucose uptake and lactate production, through the HIF1-α/PDK4 pathway. Overexpression of PDK4 demonstrated that G-Rh2 targeted the inhibition of PDK4 expression, thereby restoring mitochondrial function, promoting reactive oxygen species (ROS) accumulation, and inducing apoptosis. When combined with sodium dichloroacetate, a PDK inhibitor, it complemented the inhibitory capacity of PDKs, acting synergistically as a detoxifier.

**Conclusion:**

G-Rh2 could target and down-regulate the expression of HIF-1α, resulting in decreased expression of glycolytic enzymes and inhibition of aerobic glycolysis in tumors. Additionally, by directly targeting mitochondrial PDK, it elevated mitochondrial oxidative phosphorylation and enhanced ROS accumulation, thereby promoting tumor cells to undergo normal apoptotic processes.

## Introduction

Lung cancer, the most common cause of cancer-associated deaths worldwide (Hirsch et al. 2017), accounts for 85% of non-small cell lung cancer (NSCLC). Despite many advances within the treatment of NSCLC in the past two decades, the overall cure and survival rates are relatively low (Wang et al. 2021; Miller and Hanna 2021). Currently, the main treatments for NSCLC have involved surgical treatment, postoperative radiotherapy, and targeted therapy, but all are ineffective. Aerobic glycolysis caused by an altered tumor microenvironment is an important factor contributing to malignant tumor progression (Chu et al. 2016; Long and Suresh 2020). Thus, new adjuvant treatment strategies are urgently needed. Currently, the advancement of Chinese medicine is providing novel insights into the treatment of lung cancer. In recent years, various forms of complementary/alternative medicine have been used to treat cancer, among which traditional Chinese medicine has been widely accepted as the dominant form of complementary and alternative therapy (Xiang et al. 2019). Some herbal medicines are now being widely studied due to their low toxicity and high effectiveness.

Ginseng, one of the oldest and most widely known herbal medicines, has long been used as a tonic to enhance immunity and slow down aging. Ginsenosides are the active ingredients found in Chinese ginseng. Previous studies have indicated that ginsenoside Rh2 (G-Rh2) can reduce neurological damage in neurological diseases (Chen et al. 2022), possess anti-inflammatory activity against rheumatoid arthritis (Tang et al. 2021), anti-diabetic (Alolga et al. 2020) and anti-fatigue (Luo et al. 2019) properties, anti-arrhythmic effects in cardiovascular diseases (Sun et al. 2016), and antitumor effects (Xiaodan and Ying 2022; Li et al. 2020a, b; Man et al. 2021). G-Rh2, a major active component of ginsenosides, has shown promising advantages in cancer treatment (Li et al. 2020a, b). For example, G-Rh2 induces apoptosis in breast cancer cells by upregulating endoplasmic reticulum stress (Liu et al. 2022); inhibits the growth of liver cancer cells (Chen et al. 2021), and triggers apoptosis in colorectal cancer cells through activation of the P53 pathway (Li et al. 2011). G-Rh2 exists in two structures: S- and R-type. The 20(S)-Rh2 monomer, which is the primary component isolated from ginseng, has demonstrated its major effect on tumor therapy (Dong et al. 2011). Therefore, this study focused on 20(S)-Rh2. Since the specific mechanism of action of G-Rh2 in NSCLC was unclear, this study was aimed to investigate how G-Rh2 inhibited malignant progression in NSCLC.

In the presence of oxygen, tumors produce adenosine triphosphate (ATP) through glycolysis instead of mitochondrial oxidative phosphorylation, a phenomenon known as the “Warburg effect” (Vander Heiden et al. 2009). This metabolic process can result in the production of large amounts of lactate. Tumor cells gain advantages in survival and proliferation within the tumor microenvironment by undergoing metabolic reprogramming (Hsu and Sabatini 2008). On the other hand, normal cells use glycolysis under aerobic conditions to convert glucose into pyruvate, which is further metabolized in mitochondria through the tricarboxylic acid cycle (TCA) and oxidative phosphorylation, ultimately producing ATP (Abdel-Wahab et al. 2019). Sodium dichloroacetate (DCA) serves as an inhibitor of pyruvate dehydrogenase kinase (PDKs) (Semba et al. 2016; Khan et al. 2017; Fujiwara et al. 2013), which stimulates the process of mitochondrial oxidative phosphorylation by inhibiting PDK and activating the pyruvate dehydrogenase complex (PDC) (Zhou et al. 2015). Currently, DCA has been used in clinical practice as an anticancer drug targeting glycolysis(Pathania et al. 2009). However, due to the hepatotoxicity (Stacpoole et al. 2008) and neurotoxicity (Stacpoole 1989; Schaefer 2006; Yount et al. 1982; Spruijt et al. 2001) associated with high doses of dichloroacetate, it is not suitable for long-term clinical use. Therefore, finding a way to minimize the dosage of DCA while simultaneously increasing its inhibitory effect on tumor glycolysis and enhancing mitochondrial function is necessary.

The hypoxia-inducible factor HIF1-α is one of the major drivers of glucose metabolism in cancer cells. It regulates metabolic energy shifts by promoting the expression of several glycolytic enzymes (Zheng et al. 2021; Lai et al. 2013). HIF1-α inhibits pyruvate dehydrogenase (PDH) activity, which affects mitochondrial function by inducing the expression of PDK. This, in turn, promotes the production of lactic acid and the ability of aerobic glycolysis. Changes in PDK4, a key enzyme in both aerobic glycolysis and mitochondrial glucose oxidation, directly impact mitochondrial function as well (Leclerc et al. 2017; Kinnaird et al. 2016). As a downstream signaling molecule of HIF1-α, the role of HIF1-α/PDK4 in glycolysis and mitochondrial function is critical (Leclerc et al. 2017; Lu et al. 2021; Li et al. 2023).

Because ginseng supports the body’s positive energy, ginsenosides Rh2 might potentially induce a reversion to normal oxidative phosphorylation by inhibiting aerobic glycolysis in tumors. However, further exploration is needed to understand the potential mechanism. Therefore, the objective of the current study was to clarify the mechanisms underlying the effects of G-Rh2 on aerobic glycolysis and oxidative phosphorylation in NSCLC.

Our preliminary research has found that G-Rh2 can potentially serve as an inhibitor of PDK4, thereby inhibiting tumor glycolysis, promoting mitochondrial oxidative phosphorylation, contributing to the benign transformation of tumors, and inducing tumors to enter the normal apoptotic program. Additionally, when combined with DCA, it showed the advantage of increasing efficacy and reducing toxicity. These findings provided evidence that tonic herbs did not directly kill tumors and might present a novel approach in clinical tumor therapy.

## Materials and methods

### Cell culture

The authenticated human lung adenocarcinoma cell lines A549 and PC9 were obtained from the Type Culture collection of the Chinese Academy of Sciences and were stored in the Shanghai Key Laboratory of Molecular Imaging. The A549 cell line was cultured at 37 ℃ under 5% CO_2_ in Dulbecco’s modified Eagle medium (DMEM) supplemented with 10% fetal bovine serum (FBS) (Gibco) and 1% penicillin-streptomycin solution (Gibco). The PC9 cell line was cultured at 37 ℃ under 5% CO_2_ in RPMI-1640 supplemented with 10% FBS (Gibco) and 1% penicillin-streptomycin solution (Gibco).

### Cell proliferation assays

Cell proliferation viability was assessed using a CCK-8 assay kit. Cells were seeded at a density of 5.0 × 10^5^ cells/well in a 96-well plate and incubated at 37 ℃ for 24 h to allow adhesion to the plate. After treating the cells with different concentrations of G-Rh2 for 24 h, 10% cell counting kit-8 (CCK-8) solution was added and incubated for 1 h at 37 ℃. The optical density was measured at 450 nm using a microplate reader to determine the IC50 value. Subsequently, the proliferation rate was determined after G-Rh2 treatment.

### Ethynyl deoxyuridine (EdU) analysis

Cell proliferation viability was assessed using an EdU cell proliferation assay kit (UE, China). Cells were seeded at a density of 3.0 × 10^5^ cells/well in a 24-well plate and incubated at 37 ℃ for 24 h to allow adhesion to the plate. Subsequently, the cells were treated with different concentrations of G-Rh2 for 24 h. Cells were labeled with EdU reagent and incubated for 2 h, fixed in 4% paraformaldehyde, permeabilized with 0.1% Triton X-100, and stained with Hoechst 33,342. Images were obtained using an inverted fluorescence microscope (Olympus) after sample preparation. The proliferation rate was determined by calculating the ratio of EdU-positive cells to the total number of Hoechst 33,342-positive cells.

### Colony formation assay

Cell proliferation viability was assessed using colony formation assay. Cells were seeded at a density of 1.0 × 10^3^ cells/well in a 12-well plate and incubated at 37 ℃ for 24 h to allow adhesion to the plate. Subsequently, the cells were treated with different concentrations of G-Rh2 for 24 h. The medium was replaced with normal medium, and the cells were cultured for 1 week. The cells were then fixed in 4% paraformaldehyde, stained with 1% crystal violet, and photographed. The colony formation rate was calculated as (clone numbers/plated cell numbers) × 100%.

### Apoptosis and cell cycle assays

Apoptosis and the cell cycle were analyzed using flow cytometry. Cells were seeded at a density of 3.0 × 10^5^ cells/well in a 12-well plate and incubated at 37℃ for 24 h to allow adhesion. Then, they were treated with different concentrations of G-Rh2 for 24 h. Detection was performed using an apoptosis detection kit. The cells were stained with an Annexin V-fluorescein isothiocyanate conjugate and propidium iodide at room temperature for 15 min in the dark. The apoptotic rate was determined using a NovoCyte 2000 Flow Cytometer (Agilent Technologies, Inc, CA, USA).

Cell cycle detection was performed using a cell cycle detection kit (Beyotime, China). Cells were fixed in 70% ethanol for 30 min at 4℃ and stained with propidium iodide in a water bath maintained at 37℃, away from light. Subsequently, the apoptotic rate was determined using a NovoCyte 2000 Flow Cytometer (Agilent Technologies, Inc, CA, USA).

### Cell migration assays

Cell migration ability was assessed using transwell assays. Cells were seeded at a density of 5.0 × 10^4^ cells/well in the upper chamber of a transwell culture insert (8 μm pore size; Corning, NY, USA), and then treated with different concentrations of G-Rh2 for 24 h. The lower chamber was filled with DMEM supplemented with 15% FBS. After incubation at 37℃ for 24 h to allow adhesion and migration, the cells were fixed in 4% paraformaldehyde and stained with 1% crystal violet. Subsequently, the cells on the upper surface of the membrane were removed using cotton swabs, and images were captured using an inverted fluorescence microscope (Olympus).

### Wound healing assays

Cell migration ability was assessed using wound healing assays. Cells were seeded at a density of 4.0 × 10^5^ cells/well in a six-well plate, and incubated at 37℃ for 24 h until they reached 100% confluence. The surface was then scratched using a 200 µl pipette tip, and the cells were treated with various concentrations of G-Rh2 for 24–48 h. Wound images were taken at 0, 24, or 48 h of incubation using an inverted fluorescence microscope (Olympus).

### RNA isolation and quantitative reverse transcription-polymerase chain reaction (RT-qPCR)

The primers utilized for qPCR were as follows: GLUT1:5’-TTGGCTCCCTGCAGTTTGGC-‘3(Forward)and5’-CCCCATAGCGGTGGACCCAT-‘3;LDHA:5’-ACGTCAGCAAGAGGGAGAAA-‘3(Forward)and5’-CGCTTCCAATAACACGGTTT-‘3;HIF-1α:5’-GCTGGCCCCAGCCGCTGGAG-‘3(Forward)and5’-GAGTGCAGGGTCAGCACTAC-‘3;PGK1:5’-CCACTTGCTGTGCCAAATGGA-‘3(Forward)and5’-GAAGGACTTTACCTTCCAGGA-‘3;PKM2:5’-ATGTCGAAGCCCCATAGTGAA-‘3(Forward)and5’-TGGGTGGTGAATCAATGTCCA-‘3;PDK4:5’-GCAGTGGTCCAAGATGCCTT-‘3(Forward)and5’-GTTCAACTGTTGCCCGCATT-‘3;C-MYC:5’-TGGAACTTACAACACCCG-‘3(Forward)and 5’-CCTCGTCGCAGTAGAAAT-‘3;β-actin:5’-CATGTACGTTGCTATCCAGGC-‘3(Forward)and5’-CTCCTTAATGTCACGCACGAT-‘3.

### Western blot assays

Cells were seeded at a density of 4.0 × 10^5^ cells/well in a six-well plate and incubated at 37℃ for 24 h to allow adherence. They were then treated with different concentrations of G-Rh2 for 24 h. Protein samples were obtained using ristocetin-induced platelet aggregation (RIPA) lysis buffer supplemented with phenylmethanesulfonyl fluoride, and the lysates were centrifuged at 12,000 rpm at 4℃ for 12 min. The protein concentration was determined using a bicinchoninic acid assay (BCA) kit (Yeasen, China). Next, the protein was precipitated by adding 5× protein loading buffer and denatured in a metal bath at 100 °C for 5 minutes. Proteins were separated using 7.5% and 12.5% sodium dodecyl sulfate polyacrylamide gel electrophoresis (SDS-PAGE) gels, and then transferred onto polyvinylidene fluoride (PVDF) membranes (Millipore). The membranes were blocked with 5% skim milk for 1 h. Subsequently, primary antibodies were incubated overnight to bind to the antigens, followed by the addition of secondary antibodies and incubation for 1 h to bind to the primary antibodies. ECL luminescence detection solution (Vazyme) was added for simultaneous exposure and development. The following primary antibodies were used to assess protein expression: CCNA2 (1:2000), N-cadherin (1:1000), E-cadherin (1:1000), HIF1-α (1:2000), and VEGFA (1:1000) from ZEN Bioscience (China); PDK4 (1:2000) from Abcam (UK); beta-tubulin (1:2000) and β-actin (1:50000) from Proteintech (China); HK2 (1:2000), LDHA (1:2000), PKFB2 (1:2000), and PKM2 (1:2000) from ABclonal (US). The intensity of the bands was analyzed using ImageJ.

### Glucose intake, pyruvic acid and lactification assays

Cells were plated at a density of 4.0 × 10^5^ cells/well in a six-well plate, incubated at 37℃ for 24 h to allow adherence, and then treated with different concentrations of G-Rh2 for 24 h. Subsequently, the cells were cultured in DMEM without FBS for serum starvation. The samples were assayed using a glucose detection kit (Sigma), lactate detection kit (Nanjing Jiancheng Bio), and pyruvate content detection kit (Solarbio, China). After adding the detection reagent, the optical density values were measured at 540 nm, 530 nm, and 520 nm using a microplate reader. A standard curve was generated by analyzing different standard dilutions, and the resulting OD values were utilized to determine glucose intake, lactification, and pyruvic acid production.

### ATP content assays

Cells were plated at a density of 4.0 × 10^5^ cells per well in a six-well plate. They were incubated at 37 ℃ for 24 h to allow adherence and then treated with different concentrations of G-Rh2 for 24 h. Afterward, 200 µl of lysate was added to each well of a six-well plate and centrifuged at 12,000 g for 5 minutes at 4 ºC. The supernatant was retained, and the ATP content was detected using an ATP assay kit (Beyotime, China). The RLU value was measured using a luminometer.

### Reactive oxygen species (ROS) assays

Cells were plated at a density of 3.0 × 10^5^ cells per well in a six-well plate, incubated at 37 ℃ for 24 h to allow adhesion and subsequently treated with different concentrations of G-Rh2 for 24 h. The levels of ROS were detected using a ROS detection kit (UE, China). The DCFH-DA probe was diluted with serum-free medium, and the cells were further incubated in a cell incubator at 37 °C for 30 min in the dark. The fluorescence intensity was then measured using a NovoCyte 2000 Flow Cytometer (Agilent Technologies, Inc, CA, USA) or captured using a fluorescence microscope (Leica, US).

### γ-count assays

Cells were plated at a density of 4.0 × 10^5^ cells per well in a 12-well plate, incubated at 37℃ for 24 h to allow adhesion and then treated with different concentrations of G-Rh2 for 24 h. Subsequently, ^18^FDG-PBS solution (4 µCi/ml) was added to each well and incubated at 37℃ for 1 h. Next, a 0.5 M NaOH solution was added to lyse the cells, after which they were placed in the detector tube. CPM values were measured using a γ-counter and standardized according to the cell counting method to analyze the experimental results.

### PET-CT assays

Nude mice (4–6 weeks old, weighing 15–20 g) that had been subcutaneously transplanted with A549 cells were treated with G-Rh2. The treatment involved intraperitoneal injection of 20 mg/kg every other day for a duration of 2 weeks. A dose of 2 mCi/ml was administered via tail vein injection at a volume of 100 µl per mouse. After allowing 30 min for metabolism, the mice underwent PET-CT scanning using equipment from Mediso in Hungary. The region of interest (ROI) for the tumor was then delineated, and the maximum standardized uptake value (SUVmax) was calculated to analyze the experimental results.

### Tumor mouse model establishment

BALB/c nude mice (4–6 weeks old, weighing 15–20 g) were purchased from Shanghai Jihui Laboratory Animal Care Co. Ltd. This experiment was approved by The Animal Ethics Committee of Shanghai University of Medicine and Health Science.

A subcutaneous tumor model in nude mice was established by injecting 100 µl of PBS solution containing 5 × 10^6^ A549 cells into the right upper limb. After 1 week, the mice were divided into two groups: (a) the control group receiving 100 µl of PBS solution, and (b) the G-Rh2 treatment group receiving a dose of 20 mg/kg body weight. Tumor volume and mouse weight were measured every other day.

To establish a model of tail vein metastasis, luciferase-labeled A549 cells (3 × 10^5^ in 100 µl of PBS) were injected into the tail vein. Two weeks after the injection, G-Rh2 (intraperitoneal injection at a dose of 20 mg/kg, every other day) was administered for 2 weeks. The lung metastasis of tumor cells was then detected by measuring the fluorescence signal in the lungs using an animal bioluminescence imaging system 15 min after intraperitoneal injection of D-fluorescein sodium salt. The number of metastatic foci in lung tissues was determined through hematoxylin and eosin (HE) staining.

To establish the lymphatic metastasis model, luciferase-labeled A549 cells (3 × 10^5^ in 100 µl of PBS solution) were injected into the paw pads of nude mice. Treatment with G-Rh2 at a dose of 20 mg/kg (intraperitoneal injection, every other day for a total of 14 days) was initiated 2 weeks after the injection. Two weeks later, the lymphatic metastasis of tumor cells was detected by measuring the fluorescence signal in the lower leg using an animal bioluminescence imaging system. This measurement was taken 15 min after intraperitoneal injection of D-fluorescein sodium salt, allowing us to determine the number of metastatic tumor cells in the plantar region.

### Proteomic analysis

A549 cells were seeded at a density of 2.0 × 10^6^ cells/well in a 10 cm dish and incubated at 37℃ for 24 h to allow adherence. After that, the cells were either treated with G-Rh2 for 24 h or left untreated. Subsequently, the cells were lysed using RIPA buffer containing 1mM phenylmethanesulfonyl fluoride. The protein concentration was determined using a BCA kit (Yeasen, China). The protein was stored at -80℃ until further experiments. For total protein extraction, 250 µg of protein was obtained and then reduced with 10 mM DTT at 37℃ for 1 h. Subsequently, it was alkylated with 50 mM IAA at 25℃ in the dark for 1 h. Next, 1.5 mL of prechilled (-20℃) 100% acetone was added, and the samples were centrifuged at 14,000 g three times for 15 min to remove impurities. The samples were then diluted with 500 µL NH_4_HCO_3_. Peptides were desalted with SPE C18 cartridges (Thermo Fisher Scientific) and lyophilized under vacuum prior to nano-HPLC-MS/MS analyses. Three biological replicates were analyzed to evaluate the reliability of protein identification, reproducibility, and accuracy.

### Immunohistochemical analysis

The tumors from both the control and ginsenoside-treated groups were dissected. The tumor tissue was then embedded in paraffin and cut into approximately 5 μm thick sections. These sections were deparaffinized and fixed in 4% paraformaldehyde. Subsequently, they were equilibrated with an equilibration buffer and incubated at 37 °C for 60 min to allow for terminal reactions. To block the endogenous peroxidase, 3,3’-diaminobenzidine (DAB) was used for color development. The slides were sealed with 100% glycerin, and the stained tissue was observed and photographed under a microscope.

### Immunofluorescence analysis

The dissected lymph node tissues were fixed using 4% paraformaldehyde, sectioned, and placed in a blocking solution to prevent non-specific binding. Then, the tissues were permeabilized and blocked. The primary antibody was incubated overnight at 4 °C, followed by incubation with the secondary antibody at room temperature in the dark. The slides were counterstained with DAPI and sealed using an anti-quenching agent. Finally, the staining was photographed under a fluorescence microscope.

### Extracellular acid ratio (ECAR) and oxygen consumption rate (OCR) analysis

Drug-treated A549 cells were incubated overnight for 24 h at a density of 15,000 cells per well. The cells were spread flat on Seahorse XFe96 Cell Culture Microplates (Agilent, Palo Alto, CA, USA) using an 80-µl cell suspension. Probe plates (Seahorse XFe96 Flux Assay Kit) were hydrated by incubating them with a hydration standard solution (XF Calibrant) at 37 °C overnight. Before the experiment, the medium was replaced with an assay solution (DMEM with 10 mM glucose, 1 mM pyruvate, and 2 mM glutamine, pH 7.4, Agilent). The cells were then incubated in a non-CO_2_ incubator for 1 h at 37 °C prior to the start of the assay. The assay was conducted using a Seahorse XF Glycolytic Rate assay Kit (Agilent) and Seahorse XF Cell Mito Stress Test Kit (Agilent) as per the manufacturer’s instructions. Rotenone/antimycin A (0.5 µM), 2-DG (50 mM), oligomycin (1 µM), FCCP (0.5 µM), and rotenone/antimycin A (0.5 µM) were added to the specific dosing wells following the manufacturer’s instructions. The OCR and ECAR data were measured and plotted using Seahorse XF96 software.

### Statistical analysis

All results were statistically analyzed using GraphPad Prism (GraphPad Software, Inc., CA, USA). T-tests were used for comparisons between two groups, and one-way analysis of variance (ANOVA) was used for comparisons among three or more groups. The data are presented as mean ± standard deviation (SD). P-values of ≤ 0.05 were considered statistically significant.

## Results

### G-Rh2 inhibited tumor cell proliferation and promotes apoptosis in vitro

**Fig. 1:**
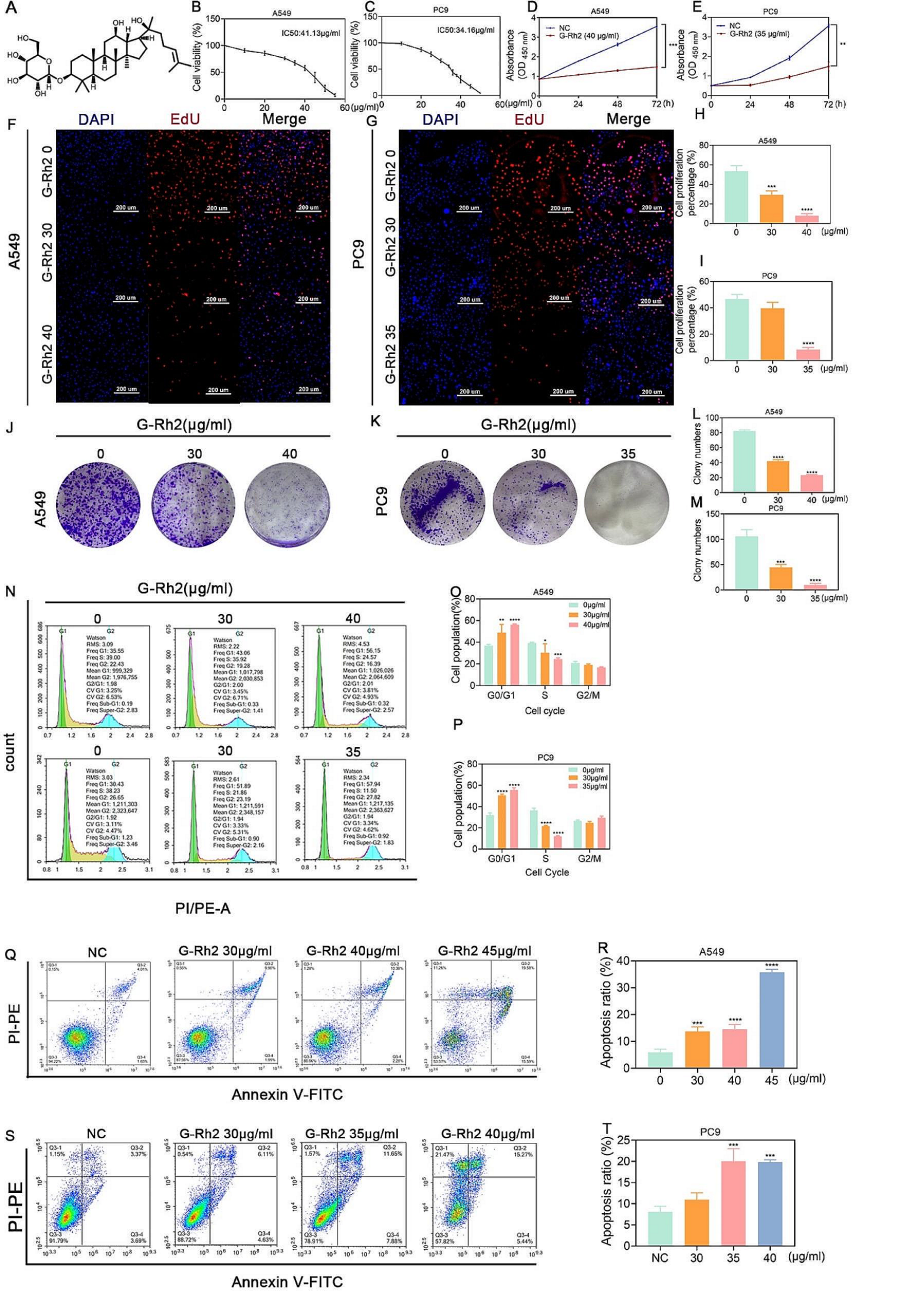
G-Rh2 inhibited tumor cell proliferation and promoted apoptosis in vitro. (**A**) Chemical structure of G-Rh2. (**B, C**) Viability of human A549 (**B**) and PC9 (**C**) cells assessed by CCK8 assays after 24-hours treatment with different concentrations of G-Rh2. (**D, E**) Effects of G-Rh2 on A549 (**D**) and PC9 (**E**) cell proliferation analyzed through CCK8 assays after IC50 treatment for 0, 24, 48, or 72 h. Data are expressed as mean ± SD. (**F-I**) EdU detection data showing A549 and PC9 cell proliferation ability following G-Rh2 treatment at 0, 30, or 40 µg/ml for A549 cells and 0, 30, or 35 µg/ml for PC9 cells for 24 h. Data are expressed as mean ± SD. (**J-M**) Colony formation assay, showing A549 and PC9 cell proliferation after G-Rh2 treatment at 0, 30, or 40 µg/ml for A549 cells and 0, 30, or 35 µg/ml for PC9 cells. Data are expressed as mean ± SD. (**N-P**) Flow cytometry measurement of the percentages of A549 and PC9 cells in G0/G1, S, and G2/M phases. Data are presented as the mean ± SD. (**Q**) Western blot detection of the cell cycle-associated protein Cyclin A2. (**R-U**) Flow cytometry analysis showing A549 and PC9 cell apoptosis after 24-hours treatment with 0, 30, 35, 40, or 45 µg/ml G-Rh2 Data are expressed as mean ± SD. *P < 0.05, **P < 0.01, ***P < 0.001, ****P < 0.001 vs. the control group

To assess the inhibitory effects of G-Rh2 on the proliferative ability of tumor cells in vitro, we conducted proliferation assay experiments using NSCLC A549 and PC9 cells. The chemical structure formula of G-Rh2 is shown in Fig. 1A. Initially, we performed the CCK8 experimental assay, which revealed that G-Rh2 treatment for 24 h inhibited the proliferation ability of A549 and PC9 cells in a concentration-dependent manner. The IC50 values were determined as 41.13 µg/ml for A549 and 34.16 µg/ml for PC9 (Fig. 1B, C). Thus, the concentration of G-Rh2 at 40 µg/ml for A549 and at 35 µg/ml for PC9 was selected for subsequent experiments. G-Rh2 treatment was performed for 0, 24, 48, or 72 h at the abovementioned concentrations, while the control group received vehicle alone. The data presented in Fig. 1D and E confirmed that G-Rh2 inhibited the proliferative ability of NSCLC cells in a time-dependent manner. EdU assays further confirmed that G-Rh2 significantly inhibited NSCLC cell proliferation, as evidenced by reduced numbers of EdU-positive cells with increasing concentrations of G-Rh2 (Fig. 1F-I). Moreover, colony formation assay demonstrated that G-Rh2 treatment resulted in fewer cloned spots compared to the control group (Fig. 1J-M). Furthermore, cell cycle detection using flow cytometry revealed that treatment with the IC50 concentration of G-Rh2 arrested the cell cycle in the G0/G1 phase (A549: 56.15% blocked; PC9: 57.94% blocked) compared to the control treatment (A549: 35.55% blocked; PC9: 30.43% blocked), as well as in the S phase (A549: 24.57% blocked; PC9: 11.5% blocked) compared to the control treatment (A549: 39% blocked; PC9: 38.23% blocked) (Fig. 1N-P). Moreover, flow cytometry apoptosis detection showed that G-Rh2 treatment for 24 h promoted apoptosis in both A549 and PC9 cells in a dose-dependent manner (Fig. 1Q-T). These results indicated that G-Rh2 inhibited tumor cell proliferation, induced G1 phase cell cycle arrest, and promoted tumor cell apoptosis in vitro.

### G-Rh2 inhibited tumor growth and promoted apoptosis in vivo

**Fig. 2:**
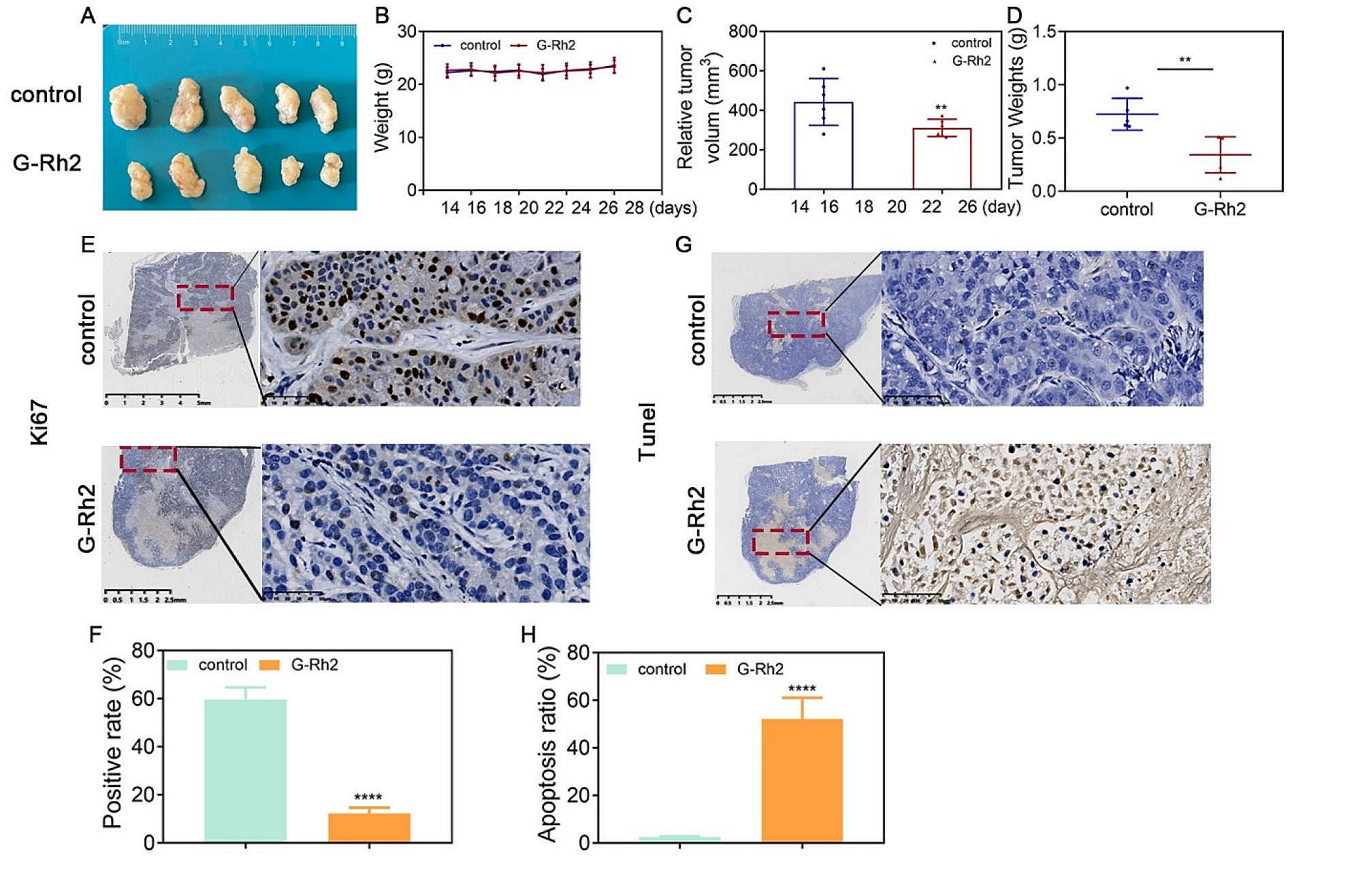
G-Rh2 inhibited tumor growth and promoted apoptosis in vivo. (**A**) Representative images of A549 tumor formation in the xenografts of nude mice. (**B**) Measurement of mouse weights after G-Rh2 treatment for 14 days. (**C**) Summary of tumor volumes recorded every 2 days. Data are expressed as mean ± SD. (**D**) Determination of tumor weights 14 days after intraperitoneal injection of G-Rh2.Data are expressed as mean ± SD. (**E-H**) Immunohistochemical analysis showing the percentages of Ki67-positive and TUNEL-positive cells. Data are expressed as mean ± SD. **P < 0.01, ***P < 0.001, ****P < 0.001 vs. the control group

Considering the substantial inhibitory effect of G-Rh2 on tumor cell proliferation observed in in vitro experiments, we constructed a model of primary tumor foci to verify whether G-Rh2 also showed significant inhibition of tumor growth in animal experiments conducted in vivo. The results of the animal experiments demonstrated that intraperitoneal administration of 20 mg/kg G-Rh2 didn’t decrease the weights of the mice, thus suggesting that 20 mg/kg G-Rh2 was non-toxic. Additionally, the in vivo tumor formation experiments revealed that G-Rh2 treatment significantly suppressed tumor growth, as evidenced by reductions in both tumor volume and weight (Fig. 2A-D). Furthermore, G-Rh2 treatment resulted in a significant decrease in the expression of the tumor growth marker Ki67, as observed through immunohistochemistry. Conversely, TUNEL staining indicated that G-Rh2 promoted tumor apoptosis (Fig. 2E-H). These findings indicated that G-Rh2 obviously suppressed the growth of primary tumor foci in vivo while concurrently inducing apoptosis.

### G-Rh2 inhibited tumor cell metastasis in vitro

**Fig. 3:**
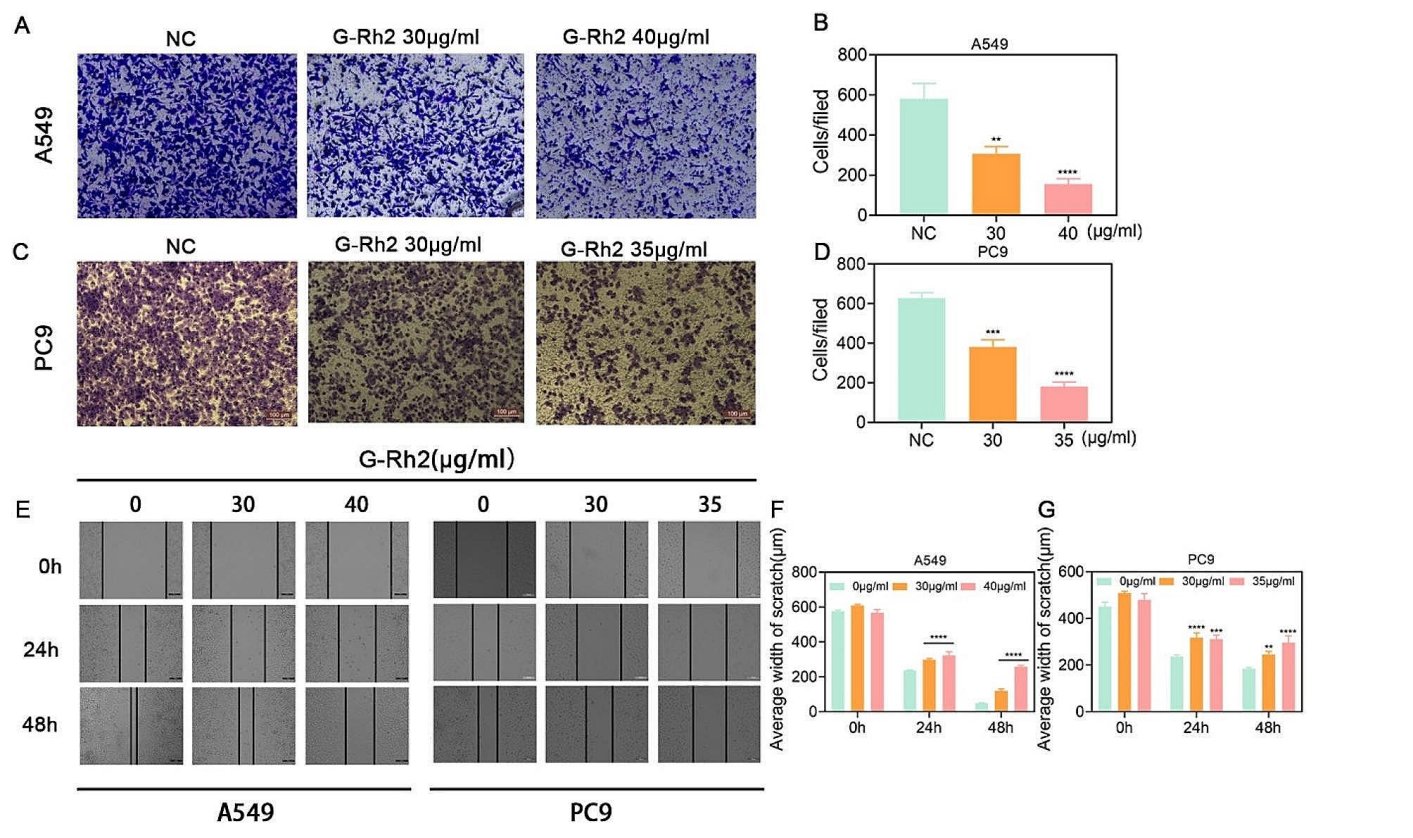
G-Rh2 inhibited tumor cell metastasis in vitro. (**A-D**) Transwell assays showing the migration ability of A549 and PC9 cells after treatment with 0, 30, 35, or 40 µg/ml G-Rh2 for 24 h. Data are expressed as mean ± SD. (**E-G**) Wound healing assays showing the invasion ability of A549 and PC9 cells after treatment with 0, 30, 35, or 40 µg/ml G-Rh2 for 0, 24, or 48 h. Data are expressed as mean ± SD. ***P < 0.001, ****P < 0.001 vs. the control group

To examine the potential inhibitory effect of G-Rh2 on the migratory ability of tumor cells in vitro, we performed assays using transwell and wound healing assays. The transwell assays confirmed the inhibitory effect of G-Rh2 on the migration of NSCLC cells (Fig. 3A-D). Similarly, the wound healing assays verified that G-Rh2 restrained the rate of wound healing, as indicated by the measured widths of the wounds (Fig. 3E-G). These results demonstrated that G-Rh2 inhibited both invasion and migration of NSCLC cells.

### G-Rh2 inhibited distal tumor metastasis in vivo

**Fig. 4:**
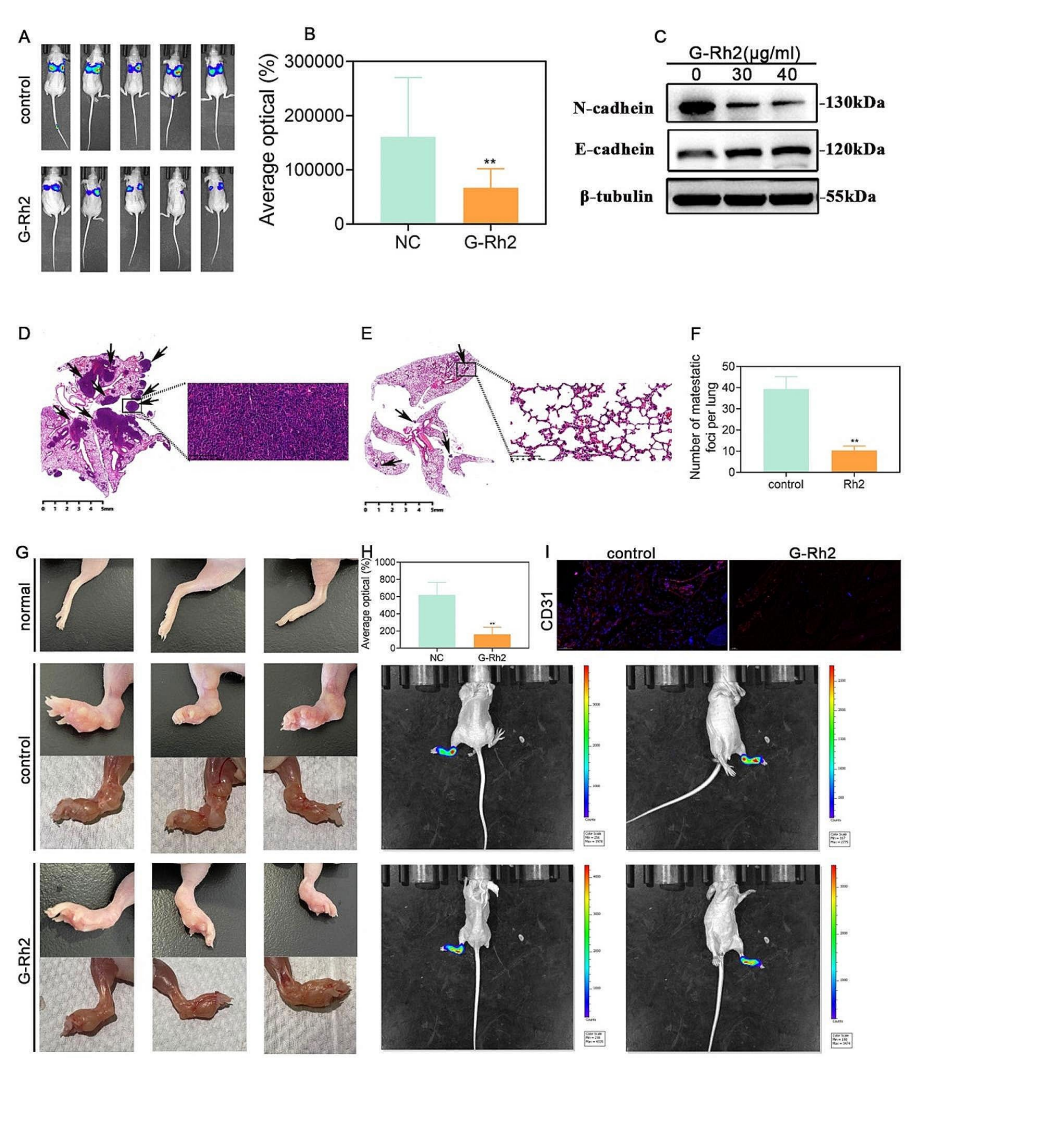
G-Rh2 inhibited distal tumor metastasis in vivo. (**A, B**) Live imaging illustrating the effects of G-Rh2 on A549 cell metastasis 14 days after tail vein injection. (**C**) Differential expression of epithelial–mesenchymal transition associated proteins detected by western blot assays. (**D-F**) The number of metastatic foci in lung tissues, calculated based on HE staining. Data are expressed as means ± SD. (**G-I**) Representative images showing A549 tumor invasion in a plantar lymphatic metastasis nude mouse model, and live imaging and immunofluorescence detection of CD31 expression showing the effect of G-Rh2 on A549 tumor invasion 2 weeks after tail vein injection. Data are expressed as mean ± SD. **P < 0.01, ***P < 0.001, ****P < 0.001 vs. the control group

Previous studies have demonstrated the inhibitory effects of G-Rh2 on tumor cell migration and metastasis, and we constructed a metastatic foci model to verify the impact of G-Rh2. Specifically, we constructed a mouse tail vein lung metastasis model and demonstrated that G-Rh2 obviously inhibited the invasion of NSCLC cells from the tail vein to the lungs based on the results of in vivo fluorescence intensity analysis of tail vein transfer experiments (Fig. 4A, B). Moreover, through Western blot analysis of proteins associated with epithelial mesenchymal transition (EMT), we observed that G-Rh2 treatment led to an increase in E-cadherin levels while decreasing N-cadherin levels (Fig. 4C). Additionally, HE staining demonstrated that G-Rh2 treatment significantly decreased the number of foci in lung tissues (Fig. 4D-F). These results indicated that G-Rh2 inhibited the metastasis of NSCLC by inhibiting the EMT process.

Plantar lymph metastasis model assays demonstrated that G-Rh2 inhibited tumor lymph metastasis based on an observed decrease in fluorescence intensity within the lymph nodes of the feet and legs (Fig. 4G, H). Immunofluorescence analysis showed that G-Rh2 treatment obviously inhibited the expression of CD31 (Fig. 4I), indicating that G-Rh2 inhibited tumor lymph angiogenesis. Collectively, these results confirmed the in vivo inhibition of tumor metastasis by G-Rh2.

### G-Rh2 inhibited tumor aerobic glycolysis by regulating HIF1-α /PDK4

**Fig. 5:**
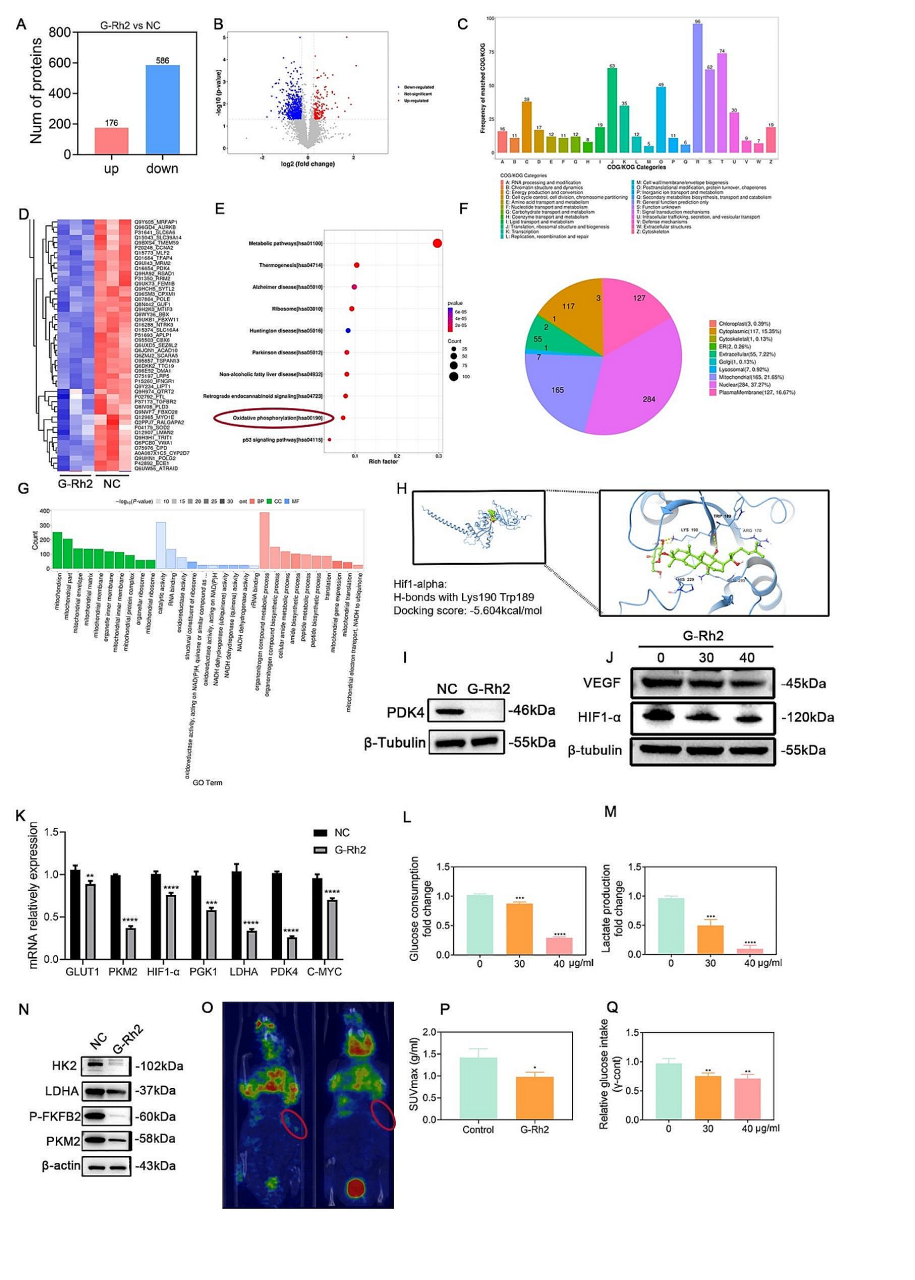
G-Rh2 inhibited tumor aerobic glycolysis by regulating HIF1-α/PDK4. (**A-B**) Volcano plots showing 176 upregulated and 586 downregulated differentially expressed proteins in A549 cells after G-Rh2 treatment compared to the control group,. (**C**) COG analysis histogram after G-Rh2 treatment, compared to the control group. (**D**) Clustered heat map showing a portion of A549 cells after G-Rh2 treatment, compared to the control group. (**E**) KEGG pathway enrichment of proteomics in A549 cells. (**F**) Subcellular location analysis after G-Rh2 treatment, compared to the control group. (**G**) GO pathway enrichment of proteomics in A549 cells. (**H**) Molecular docking modeling predicting the binding of G-Rh2 to HIF-1α. (**I**) Western blot detection of PDK4 protein expression. (**J**)Western blot detection of HIF1-α and VEGF protein expression. (**K**) RT-qPCR detection of downstream genes of HIF1-α. (**L**) Glucose intake in A549 cells after G-Rh2 treatment. (**M**) Lactification in A549 cells after G-Rh2 treatment. (**N**) Western blot detection of glycolytic protein expression. (**O, P**) PET-CT detection of the concentration of ^18^FDG in A549 tumors, revealing glucose intake after G-Rh2 treatment in xenografts of nude mice. (**Q**) γ-count data showing glucose intake of A549 tumors after G-Rh2 treatment in xenografts of nude mice. Data are expressed as mean ± SD.**P < 0.01, ***P < 0.001, ****P < 0.001 vs. the control group

To verify the relationship between G-Rh2 and HIF1-α, we conducted proteomic analysis to identify the differentially expressed proteins in A549 cells before and after G-Rh2 treatment. The analysis identified 176 upregulated and 586 downregulated proteins in G-Rh2-treated A549 cells (Fig. 5A, B). Furthermore, clusters of orthologous genes (COG) analysis indicated that the differentially expressed genes were primarily associated with signal transduction mechanisms (Fig. 5C).

KEGG enrichment analysis confirmed that G-Rh2 treatment is associated with enrichment in the metabolic pathway and oxidative phosphorylation pathway. The heatmap showed that the differences in PDK4 proteins in the metabolic pathways were more significant (Fig. 5D, E). Subcellular analyses showed that, apart from unclear enrichment, the majority of genes re-executing their functions were predominantly located within mitochondrial structures (Fig. 5F). GO analysis (Fig. 5G) also confirmed that the differentially expressed proteins after ginsenoside treatment were mainly enriched for mitochondrial redox reactions.

KEGG enrichment analysis of the proteomic findings demonstrated that G-Rh2 inhibits the growth of NSCLC through the glucose metabolic pathway. HIF1-α is known to regulate the expression of PDK4, a pivotal enzyme during the glucose oxidation process. Inhibition of PDK4 promotes the aerobic glucose oxidation process. Molecular docking modeling predicted strong binding of G-Rh2 to HIF1-α (Fig. 5H), with H-bonds formed with Lys190 Trp189, and a docking score of -5.604 kcal/mol. Western blot assays confirmed that G-Rh2 inhibited the expression of PDK4 (Fig. 5I), and demonstrated the inhibitory effect of G-Rh2 treatment on the expressions of HIF1-α as well as VEGF in A549 cells (Fig. 5J). We examined the expression of the downstream target genes of HIF1-α. RT-qPCR results showed the inhibitory effect of G-Rh2 on the expression of its downstream target genes PKM2, GLUT1, PGK1, PDK4, and LDHA, among which the inhibitory effect of PDK4 was the most significant, indicating that G-Rh2 inhibited the HIF1-α pathway (Fig. 5K). In vitro assays of glucose intake and lactate production were performed. The G-Rh2-treated group showed significant inhibition of glucose intake as well as lactate production in NSCLC cells (Fig. 5L, M). Western blot detection proved that G-Rh2 treatment downregulated the expression of of the expression of glycolysis-related proteins HK2, LDHA, PFKFB2, and PKM2 (Fig. 5N), indicating the inhibitory impact of G-Rh2 on the glycolytic process of tumors. In vivo, G-Rh2 inhibited glucose uptake in tumor tissues, as evidenced by a comparison of the SUVmax values with the ROI regions (Fig. 5O, P). The γ-counter detected significantly inhibited glucose intake of G-Rh2-treated cells, based on a comparison of CPM values (Fig. 5Q). These results suggested that G-Rh2 regulated HIF1-α and downregulated PDK4 expression, thereby inhibiting aerobic glycolysis in NSCLC.

### G-Rh2 targeted inhibition of PDK4 expression promoted mitochondrial oxidative phosphorylation

**Fig. 6:**
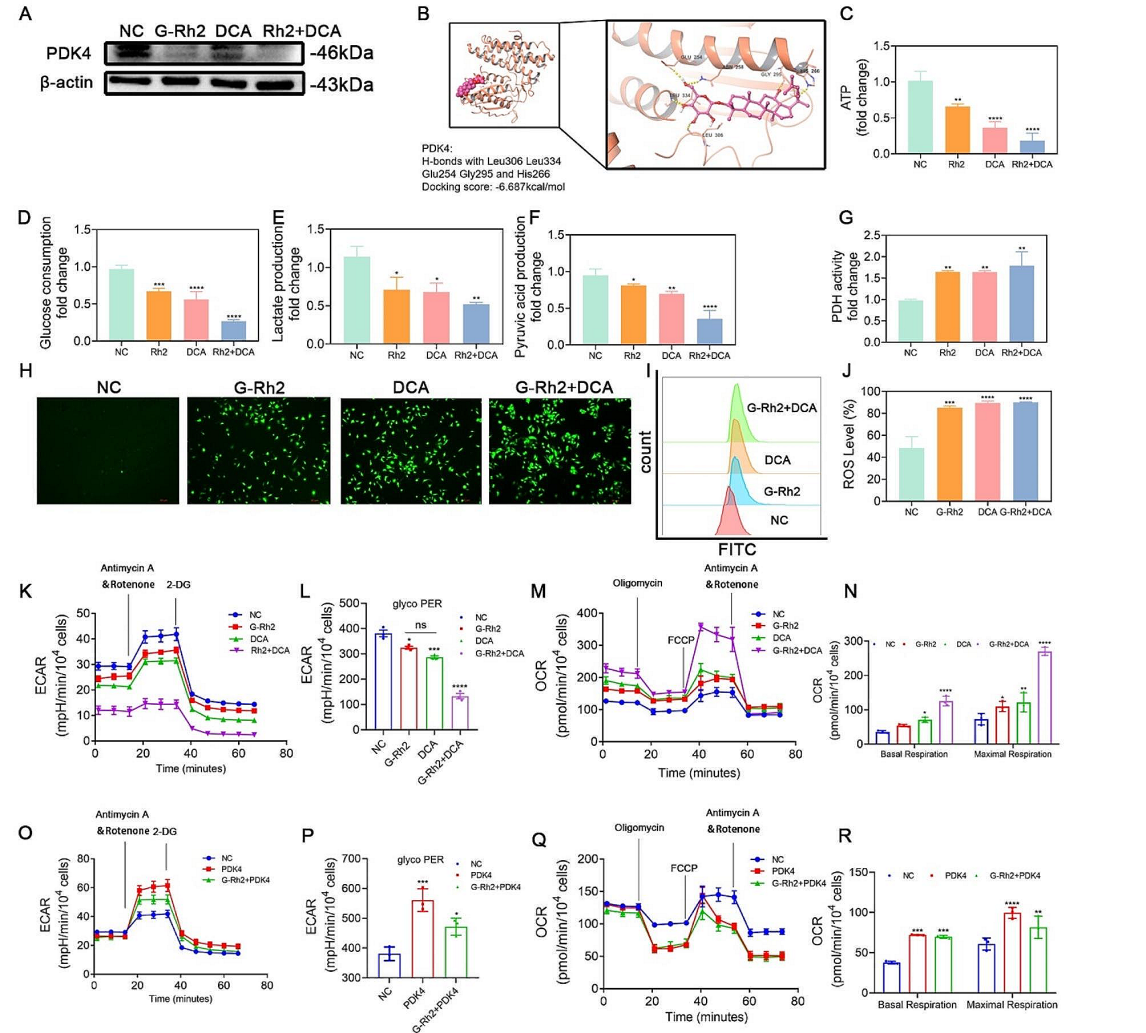
G-Rh2 targeted inhibition of PDK4 expression promoted mitochondrial oxidative phosphorylation. (**A**) Western blot detection of PDK4 protein expression following G-Rh2 and DCA treatment. (**B**) Molecular docking modeling predicts the binding of G-Rh2 to PDK4. (**C**) ATP production in A549 cells after G-Rh2 and DCA treatment. (**D**) Glucose intake in A549 cells after G-Rh2 and DCA treatment. (**E**) Lactification in A549 cells after G-Rh2 and DCA treatment. (**F**) Pyruvic acid production in A549 cells after G-Rh2 and DCA treatment. (**G**) PDH activity in A549 cells after G-Rh2 and DCA treatment. (**H-J**) Fluorescence detection and flow cytometry analysis of ROS levels in A549 cells after G-Rh2 and DCA treatment. Data are expressed as mean ± SD. (**K, L**) ECAR measured with Seahorse XF in A549 cells and A549 cells treated with G-Rh2 and DCA. 2-DG, 2-deoxyglucose. (**M, N**) OCR measured with Seahorse XF in A549 cells and A549 cells treated with G-Rh2 and DCA. FCCP, carbonyl cyanide 4-(trifluoromethoxy) phenylhydrazone. (**O, P**) ECAR measured with Seahorse XF in A549 and PDK4 overexpressing A549 cells treated with G-Rh2. (**Q, R**) OCR measured with Seahorse XF in A549 cells and PDK4 overexpressing A549 cells treated with G-Rh2. **P < 0.01, ***P < 0.001, ****P < 0.001 vs. the control group

G-Rh2 has been shown to target HIF-1α and downregulate PDK4 expression, thus inhibiting tumor glycolysis. Consequently, G-Rh2 may be a potential PDK4 inhibitor. Therefore, we selected DCA, an inhibitor of PDK, for comparison. DCA competitively inhibits all PDKs. PDKs can lead to inactivation by phosphorylation, inhibiting the entry of pyruvate into the TCA cycle and promoting aerobic glycolysis. DCA inhibits the expression of PDKs, promotes PDH activity, and catalyzes the conversion of pyruvate to acetyl-CoA, which then enters the TCA cycle and completes the oxidative phosphorylation process. Western blot analysis of PDK4 expression in A549 cells treated with G-Rh2 and DCA revealed that both substances similarly downregulated PDK4 expression (Fig. 6A). Molecular docking modeling predicted a strong binding between G-Rh2 and PDK4 (Fig. 6B), forming hydrogen bonds with Leu306, Leu334, Glu254, Gly295, and His266. The docking score was − 6.687 kcal/mol. Subsequently, in vitro assays of glucose uptake, lactate production, and pyruvic acid production in A549 cells treated with G-Rh2 and DCA revealed that both substances reduced glucose uptake, lactate production, and pyruvic acid production; promoted PDH activity; and inhibited ATP production (Fig. 6C-G). These findings indicated that G-Rh2 inhibited tumor glycolysis to a similar extent as DCA, suggesting that the inhibition of PDK4 by G-Rh2 complemented the effect of DCA. ROS levels were measured in different treatment groups and revealed that both G-Rh2 and DCA increased ROS production in A549 cells, suggesting that both treatments inhibited PDH activity by suppressing PDK4 expression, promoted oxidative phosphorylation of glucose, inhibited glycolysis, and increased ROS production (Fig. 6H-J). The bioenergetic profiles of A549 cells were examined using a Seahorse XFe96 Analyzer. The ECAR values of glycol PER in A549 cells treated with G-Rh2 and DCA were much lower in contrast with the control group, indicating that both substances inhibited the glycolytic capability of NSCLC cells (Fig. 6K, L). The basal OCR values and maximal respiration values were significantly higher in the G-Rh2 and DCA groups compared to the control group, indicating that both treatments promoted mitochondrial respiration in NSCLC cells and facilitated the oxidative phosphorylation process (Fig. 6M, N). Treatment of A549 cells and PDK4 overexpressing A549 cells with G-Rh2 revealed that PDK4 overexpression significantly increased the ECAR value of glycol PER, promoting aerobic glycolysis and reversing the inhibitory impact of G-Rh2 on A549 cells (Fig. 6O, P). Additionally, after treating A549 cells and PDK4 overexpressing A549 cells with G-Rh2, PDK4 overexpression significantly suppressed basic OCR values and maximal respiration values, which inhibited aerobic oxidative processes and reversed the ability of G-Rh2 to promote oxidative phosphorylation in A549 cells (Fig. 6Q, R). Therefore, G-Rh2 might act as a potential PDK4 inhibitor, activating PDH activity, facilitating the conversion of pyruvate to acetyl-CoA, reversing aerobic glycolysis in tumors, and promoting oxidative phosphorylation. Moreover, G-Rh2 focused on the regulation of glucose metabolism by modulating the target protein PDK4.

### G-Rh2 in combination with DCA reversed the biometabolic behavior of the tumor and reduced DCA dosage

**Fig. 7:**
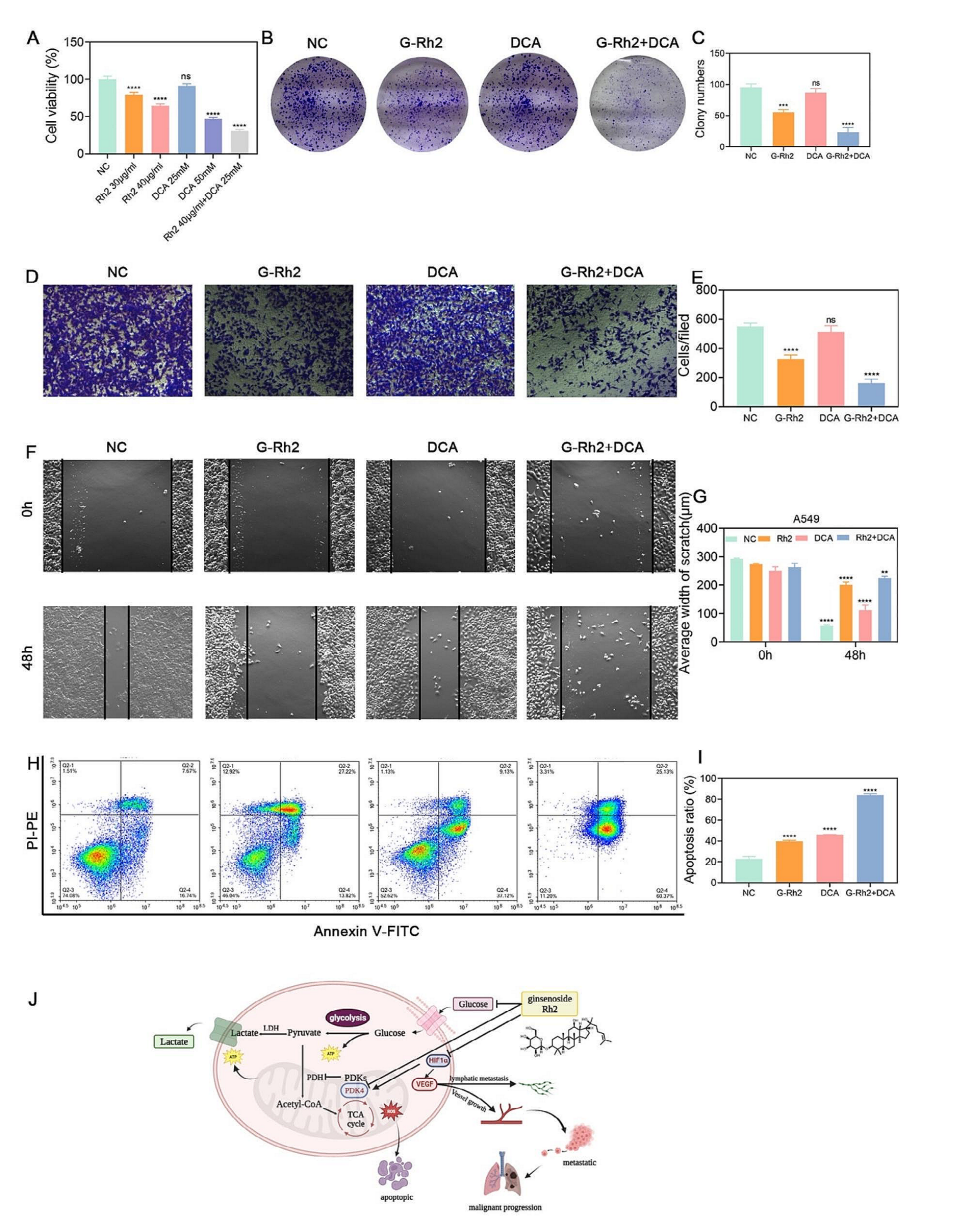
Combination of G-Rh2 with DCA reversed the tumor’s biometabolic behavior and reduced DCA dosage. (**A**) Viability of human A549 cells assessed by CCK8 assays after 24-hours treatment with different concentrations of G-Rh2 and DCA. (**B, C**) Colony formation assay demonstrating A549 cell proliferation following treatment with G-Rh2 and DCA. (**D, E**) Transwell assays measuring A549 cell migration ability after 24-hours treatment with G-Rh2 and DCA. (**F, G**) Wound healing assays indicating the inhibitory effect of G-Rh2 on the invasion ability of A549 cells after 24-hours treatment with G-Rh2 and DCA. (**H-I**) Flow cytometry analysis showing A549 cell apoptosis after treatment with G-Rh2 and DCA. (**J**) Proposed mechanism illustrating G-Rh2-induced changes in glucose metabolism in NSCLC cells. Data are expressed as mean ± SD, *p < 0.05, **p < 0.01, ***p < 0.001, ****p < 0.001 vs. the control group

After confirming that G-Rh2 may directly target PDK4 as a potential inhibitor, we chose the PDK inhibitor DCA for combination therapy. DCA is a broad-spectrum PDK inhibitor (Bonnet et al. 2007) that is not commonly used in clinical settings due to its neurotoxicity. Co-administration of G-Rh2 at the IC50 concentration with a low concentration of DCA significantly inhibited cell viability, as revealed by CCK8 detection of A549 cell proliferation (Fig. 7A). Colony formation assay further confirmed that combined treatment with G-Rh2 and DCA resulted in fewer cloned spots compared to the control group (Fig. 7B, C). When G-Rh2 at the IC50 concentration was combined with a low concentration of DCA, transwell and wound healing assays demonstrated a significant inhibition of A549 cell migration (Fig. 7D-G). Flow cytometry apoptosis detection showed that G-Rh2 and DCA treatment significantly promoted apoptosis in A549 cells (Fig. 7H, I). The above experiments demonstrated that G-Rh2, as a potential PDK4 inhibitor, could change the metabolic behavior of tumors from aerobic glycolysis to oxidative phosphorylation. Furthermore, when combined with a low dose of DCA, it could reduce the toxicity of DCA and facilitate the shift in metabolic behavior.

## Discussion

As we all know, ginseng is a high-quality traditional Chinese herb in the Wujiake family. It is widely known for its effects of tonifying vital energy, calming the mind, and improving the immune system. G-Rh2, one of the primary active components found in ginseng, has been extensively studied and shown to possess various pharmacological properties. It has demonstrated the ability to inhibit tumor progression, alleviate side effects caused by radiation and chemotherapy (Li et al. 2020a, b), and enhance immune function (Xiaodan and Ying 2022; Lee et al. 2018) in the treatment of both malignant and non-malignant diseases (Guan and Qi 2023). Several reports suggest that ginseng is widely used to treat advanced tumors (Yang et al. 2022; Xiao et al. 2019; Wang et al. 2018)In our current study, we also observed a corresponding tumor-suppressive effect of G-Rh2. It suppressed the proliferation as well as the invasion of NSCLC, and enhanced apoptosis at higher doses. However, this was not its primary function. Surprisingly, our study revealed for the first time that G-Rh2 could modulate the metabolic behavior of tumors, specifically reversing the shift from tumor aerobic glycolysis to oxidative phosphorylation, and ultimately promoting the “benignization"” of tumors. Thus, it may serve as a crucial deterrent against malignant tumor progression.

The Warburg effect, as a hallmark of cancer, means that tumors rely on glycolysis for energy, either aerobically or anaerobically (Hsu and Sabatini 2008; Liberti and Locasale 2016; Koppenol et al. 2011). Pyruvate enters the TCA cycle via PDH, and PDK facilitates the conversion of aerobic oxidation to aerobic glycolysis by inhibiting PDH activity (Leclerc et al. 2017). PDK is highly expressed in several cancers (Atas et al. 2020), which have four isoforms of PDK1-4. PDK1 is a pivotal glycolytic enzyme linked to tumor proliferation (Du et al. 2016; Deng et al. 2013) and metastasis (Dupuy et al. 2015; Zhou et al. 2019). Previous studies have shown that PDK1 can activate the PI3K/Akt/mTOR pathway to promote tumor proliferation and invasion (Jiang et al. 2021; Sambandam et al. 2019); Additionally, phosphorylation of PDK1, an upstream regulator of C-Myc, activates PLK1-MYC signaling and promotes cancer proliferation (Chinen et al. 2014). PDK1 regulation by HIF-1α promotes tumor glycolysis and malignant progression (Peng et al. 2018; Kim et al. 2006). DCA is an inhibitor of PDK1. It activates the PDC through inhibition of PDK1. The activation of PDC catalyzes pyruvate into acetyl coenzyme A, which is later converted into citrate to enter the TCA cycle and promotes the oxidative phosphorylation process in mitochondria (Zhou et al. 2015). The above demonstrates the effectiveness of targeting PDK1. PDK2 promotes multiple tumor drug resistance by inhibiting tumor mitochondrial function (Kitamura et al. 2021; Liang et al. 2020) and negatively regulating macrophage polarization(Li et al. 2017). Less research has been done in this area regarding tumors. High PDK3 expression can drive glycolysis in tumor-resistant cells (Xu et al. 2019), increasing the hypoxic response of tumor cells and promoting glycolytic processes (Li et al. 2022). PDK4 is the most widely distributed PDK subtype (Li et al. 2020a, b), an essential mitochondrial matrix enzyme in cellular energy metabolism, highly expressed in various malignant tumors (Leclerc et al. 2017). It is a key factor linking glycolysis and the TCA cycle. Targeting PDK4 to inhibit tumor glycolysis and restore mitochondrial oxidative phosphorylation function has become a hotspot for tumor therapy and an important target for clinical treatment, attracting high attention. PDK4 plays a key role in both metabolic diseases and cancer, and impaired mitochondrial function is one of the causes of several metabolic diseases. Increased activity of PDK4 in skeletal muscle inhibits glucose oxidation, thereby exacerbating blood glucose levels, and is therefore highly expressed in diabetes (Li et al. 2023). In atherosclerosis, high expression of PDK4 promotes vascular calcification (Kulkarni et al. 2012), and its absence also inhibits platelet function and arterial thrombosis (Ma et al. 2020). High expression of PDK4 in pulmonary arterial hypertension causes pulmonary artery wall cells to undergo a transition from glucose oxidation to aerobic glycolysis (Lu et al. 2021). In cancer, PDK4 is a key enzyme in aerobic glycolysis and mitochondrial oxidative reactions. Its high expression inhibits mitochondrial oxidative respiration, metabolically reprograms and promotes lactate production, which increases the glycolytic capacity of tumors, thus promoting malignant progression (Zheng et al. 2021; Li et al. 2020a, b; Flora et al. 2023; Dou et al. 2023). Currently, DCA has been used for treating mitochondrial metabolic diseases (Stacpoole et al. 2008; Barshop et al. 2004; Michelakis et al. 2017), malignant tumors (Vander Heiden 2010; Michelakis et al. 2010),etc. However, the hepatotoxicity and neurotoxicity of DCA at high doses limit its clinical use (Stacpoole 1989; Stacpoole et al. 1998; Tao et al. 2000). PDK1 and PDK4 have similarities and have been widely analyzed. Here, we found that G-Rh2, as a fostering drug, had no severe toxic effects (Guan and Qi 2023) but an inhibitory effect against the PDK4 target for the first time. It could act synergistically with lower doses of DCA to inhibit both sites of PDK, acting as a potentiator and detoxifier.

Based on the above findings, this study suggests for the first time that the vital function of G-Rh2 is to act on the PDK4 locus. The key results demonstrated that G-Rh2 could inhibit tumor glycolysis, promote oxidative phosphorylation, stimulate ROS production, and induce apoptosis. As a result, tumors changed their biological behavior due to metabolic shifts and showed a benign trend. Combining low-dose DCA with PDK reinforces its inhibitory effect and promotes the transition from glycolysis to aerobic oxidation in tumors. Previous studies have shown that G-Rh2 can inhibit the malignant progression of NSCLC through metabolic modulation of the immune environment and other pathways (Qian et al. 2019; Ma et al. 2023; Li et al. 2018). Some studies have also discovered that G-Rh2 has an impact on aerobic glycolysis and mitochondrial oxidative phosphorylation in tumors. It activates the apoptotic program of tumor cells and demonstrates a potentiating and toxicity-reducing effect when used in combination with chemotherapeutic agents (Liu et al. 2020, 2021). Compared to the toxic effects of DCA, ginseng has been applied in clinical practice for thousands of years and is an important remedy to support the body’s well-being. Our study further proved that instead of solely killing the tumors, the primary aim of Rh2 was to promote the oxidative phosphorylation function of tumors, change the behavior of tumor metabolism, promote tumors to shift to benign metabolic profile, and converge towards favorable biological behavior, ultimately leading to the activation of normal apoptotic programs. Therefore, G-Rh2 might server as a highly promising target drug with significant clinical potential. Although we have demonstrated the potential of G-Rh2 to inhibit the malignant progression of NSCLC, there has not been an in-depth study on the specific mechanism of action in regulating PDK4. We are continuing our research to identify the direct targets of G-Rh2 against tumor mitochondrial function and glucose metabolism, hoping to provide new and effective targets as well as novel ideas for clinical tumor control.

## Conclusion

This study investigated the regulatory targets and mechanisms of G-Rh2 for NSCLC. For the first time, we confirmed how G-Rh2 changed the biometabolic behavior of tumors by regulating PDK4, leading to a favorable trend towards normal apoptotic programming. We confirmed that G-Rh2 was capable of targeting and down-regulating HIF-1α’s expression, subsequently reducing the expression of PDK4. It further suppressed aerobic glycolysis, promoted mitochondrial aerobic oxidative process, and stimulated the production of ROS to promote tumor cells to enter the normal apoptotic program through PDK4 regulation. The combination of G-Rh2 with DCA, an inhibitor of PDKs, dramatically reversed the biometabolic behaviors of tumors, and further reduced the dosage of DCA, thereby decreasing the toxicity of DCA. Ginseng, known primarily as a deficiency tonic, does not mainly function through direct tumor destruction. Instead, altering the biometabolic behavior of tumors offers a novel strategy for future treatment options. The effects of G-Rh2 at the PDK4 locus suggest G-Rh2 could be a potential therapeutic agent in clinical settings. In conclusion, our results confirmed the ability of G-Rh2 to target at the PDK4 locus, providing molecular evidence for altered tumor biometabolic behavior.

## Data Availability

Not applicable.

## Abbreviations

ATP: Adenosine triphosphate
ANOVA: One-way analysis of variance
BCA: Bicinchoninic acid assay
CCK-8: Cell Counting Kit-8
DMEM: Dulbecco?s modified Eagle medium
DCA: Sodium dichloroacetate
EdU: Ethynyl deoxyuridine
ECAR: Extracellular acid ratio
EMT: Epithelial mesenchymal transition
FBS: Fetal bovine serum
G-Rh2: Ginsenoside Rh2
HE: Hematoxylin and eosin
NSCLC: Non-small cell lung cancer
OCR: Oxygen consumption rate
PDH: Pyruvate dehydrogenase
PDC: Pyruvate dehydrogenase complex
PDKs: Pyruvate dehydrogenase kinase
RIPA: Ristocetin-induced platelet aggregation
PDK4: Pyruvate dehydrogenase kinase 4
PVDF: Polyvinylidene fluoride
ROS: Reactive oxygen species
ROI: Region of interest
RT-qPCR: Quantitative reverse transcription-polymerase chain reaction
SDS-PAGE: Sodium dodecyl sulfate polyacrylamide gel electrophoresis
TCA: Tricarboxylic acid cycle
